# Achieving Thoracic Oncology data collection in Europe: a precursor study in 35 Countries

**DOI:** 10.1186/s12885-018-5009-y

**Published:** 2018-11-20

**Authors:** Anna Rich, David Baldwin, Inmaculada Alfageme, Paul Beckett, Thierry Berghmans, Stephen Brincat, Otto Burghuber, Alexandru Corlateanu, Tanja Cufer, Ronald Damhuis, Edvardas Danila, Joanna Domagala-Kulawik, Stefano Elia, Mina Gaga, Tuncay Goksel, Bogdan Grigoriu, Gunnar Hillerdal, Rudolf Maria Huber, Erik Jakobsen, Steinn Jonsson, Dragana Jovanovic, Elena Kavcova, Assia Konsoulova, Tanel Laisaar, Riitta Makitaro, Bakir Mehic, Robert Milroy, Judit Moldvay, Ross Morgan, Milda Nanushi, Marianne Paesmans, Paul Martin Putora, Miroslav Samarzija, Arnaud Scherpereel, Marc Schlesser, Jean-Paul Sculier, Jana Skrickova, Renato Sotto-Mayor, Trond-Eirik Strand, Paul Van Schil, Torsten-Gerriet Blum

**Affiliations:** 1 Department of Respiratory Medicine, Nottingham University Hospitals, City campus, Hucknall Road, Nottingham, NG5 1PB UK; 2 Respiratory medicine Department, Seville University, Seville, Spain; 3 Department of Respiratory Medicine, Derby Teaching Hospitals NHS Foundation Trust, Derby, UK; 4 Intensive Care and Thoracic Oncology, Institut Jules Bordet, Université Libre de Bruxelles, Brussels, Belgium; 5 Sir Anthony Mamo oncology centre, Mater Dei hospital, Msida, Malta; 6 Department of Respiratory and Critical Care Medicine and Ludwig Boltzmann Institute of COPD and Respiratory Epidemiology, Otto Wagner Hospital, Vienna, Austria; 7 Department of Respiratory Medicine, State University of Medicine and Pharmacy “Nicolae Testemitanu”, Chisinau, Moldova; 8 University Clinic Golnik, Medical Faculty Ljubljana, Golnik, Slovenia; 9 Department of Research, Netherlands Comprehensive Cancer Organisation (IKNL), Utrecht, The Netherlands; 10 Clinic of Infectious and Chest Diseases, Dermatovenereology and Allergology, Vilnius University, Vilnius, Lithuania; 11 Centre of Pulmonology and Allergology, Vilnius University Hospital Santariskiu Klinikos, Vilnius, Lithuania; 12 Department of Pneumonology, Medical University of Warsaw, Warsaw, Poland; 13 Department of Thoracic Surgery, University of Rome Tor Vergata, Rome, Italy; 14 7th Respiratory Medicine Department, Athens Chest Hospital, 152 Mesogion Ave Athens, 11527 Athens, Greece; 15 Department of Pulmonary Medicine, School of Medicine, Ege University, Izmir, Turkey; 16 Regional Institute of Oncology, University of Medicine and Pharmacy, Iasi, Romania; 17 Department of Respiratory Diseases, Karolinska Hospital, Stockholm, Sweden; 18 Division of Respiratory Medicine and Thoracic Oncology, University of Munich and Thoracic Oncology Centre, Munich, Germany; 19 Department of Thoracic Surgery, Odense University Hospital, Odense, Denmark; 20 Department of Medicine, Landspitali, University of Iceland, Reykjavik, Iceland; 21 University Hospital of Pulmonology, Clinical Center of Serbia, Belgrade, Serbia; 22 Clinic of Pneumology and Phthisiology, Comenius University Bratislava, Jessenius Faculty of Medicine Martin, University Hospital, Martin, Slovak Republic; 23 Medical Oncology Department, University Hospital Sveta Marina, Varna, Bulgaria; 24 Department of Thoracic Surgery, Tartu University Hospital, Tartu, Estonia; 25 Department of Internal Medicine, Respiratory Research Unit, Medical Research Center Oulu, Oulu, Finland; 26 University Hospital and University of Oulu, POB 20, 90029 Oulu, Finland; 27 Clinic of Lung Diseases and TB, Sarajevo University Clinical Centre, Sarajevo, Bosnia and Herzegovina; 28 Consultant Respiratory Physician & Chair, Scottish Lung Cancer Forum, Glasgow Royal Infirmary, Glasgow, Scotland; 29 Department of Tumor Biology, National Koranyi Institute, Semmelweis University, Budapest, Hungary; 30 Department of Respiratory Medicine, Beaumont Hospital, Dublin, 9 Ireland; 31 University of Tirana, Service of Pulmonology, Tirana, Albania; 32 Data Centre, Institut Jules Bordet, Université Libre de Bruxelles, Brussels, Belgium; 33 Department of Radiation Oncology, Kantonsspital St. Gallen, 9007 St. Gallen, Switzerland; 34 Department of Respiratory medicine, Klinički bolnički centar Zagreb, Zagreb, Croatia; 35 Pulmonary and Thoracic Oncology, Univ. Lille, Inserm, CHU Lille, U1019 – CIIL, F-59000 Lille, France; 36 Respiratory Medicine Department, Centre Hospitalier Luxembourg, Luxembourg City, Luxembourg; 37 Department Pulmonary Disease and TB, Masaryk University Faculty of Medicine & University Hospital, Brno, Czech Republic; 38 Pulmonology Service, Thoracic Department, North Lisbon Hospital Centre, Lisbon, Portugal; 39 Department of Registration, Cancer Registry of Norway, Oslo, Norway; 40 Department of Thoracic and Vascular Surgery, Antwerp University Hospital, Edegem, Antwerp Belgium; 41 Klinik für Pneumologie, Lungenklinik Heckeshorn, HELIOS Klinikum Emil von Behring, Berlin, Germany

**Keywords:** Lung Cancer, Epidemiology, Audit, Data collection, Datasets

## Abstract

**Background:**

A minority of European countries have participated in international comparisons with high level data on lung cancer. However, the nature and extent of data collection across the continent is simply unknown, and without accurate data collection it is not possible to compare practice and set benchmarks to which lung cancer services can aspire.

**Methods:**

Using an established network of lung cancer specialists in 37 European countries, a survey was distributed in December 2014. The results relate to current practice in each country at the time, early 2015. The results were compiled and then verified with co-authors over the following months.

**Results:**

Thirty-five completed surveys were received which describe a range of current practice for lung cancer data collection. Thirty countries have data collection at the national level, but this is not so in Albania, Bosnia-Herzegovina, Italy, Spain and Switzerland. Data collection varied from paper records with no survival analysis, to well-established electronic databases with links to census data and survival analyses.

**Conclusion:**

Using a network of committed clinicians, we have gathered validated comparative data reporting an observed difference in data collection mechanisms across Europe. We have identified the need to develop a well-designed dataset, whilst acknowledging what is feasible within each country, and aspiring to collect high quality data for clinical research.

**Electronic supplementary material:**

The online version of this article (10.1186/s12885-018-5009-y) contains supplementary material, which is available to authorized users.

## Background

Whilst Europe contains one eighth of the world’s population, it accounts for a quarter of all reported cases of cancer [1]. Lung cancer remains the commonest cause of death from cancer in both men and women across Europe and has one of the worst prognoses of all cancers [2]. It constitutes an enormous health burden across the continent and its incidence corresponds to the historic tobacco smoking rates. In the absence of a therapeutic breakthrough, the cancer community must ensure that it implements current best practice as effectively as possible. Our priorities should be to improve outcome by: reducing smoking prevalence through public health campaigns, improving early diagnosis, eradicating inequality in access to investigations and treatment, assuring access to novel therapies and reducing the number of patients who present via the emergency department when their prognosis is much worse [3].

Several publications have documented a variation in outcome from lung cancer across Europe in the last 15 years [2, 4], but there has been minimal attention to correlating these differences in outcome with clinical practice and clinical resources. It is not clear how much this variation depends on the historical, cultural and political background of a country. The number of independent countries in Europe has significantly increased in the last twenty-five years, and there is a self-evident wide variation in population size, economic stability and healthcare infrastructure. As an example of the diverse healthcare infrastructure in Europe, Table 1 illustrates the variation in access to primary care which was recorded in 2011 [5]. Without this information, it is difficult to make comparisons between countries, and impossible to learn from different practices and identify the key elements within the whole pathway that limit the implementation of an optimal standard of care in each country.

**Table 1:** Access to primary care (survey from 2011 part of ERS taskforce) [5]

Country	Remarks
***“free for everyone”***	
Austria	
Belarus	
Denmark	
Hungary	
Ireland	For those individuals with a ‘medical card’.
Italy	
Kyrgyzstan	
Lithuania	
Malta	
Poland	
Portugal	
Spain	
Turkey	
Ukraine	
United Kingdom	
*“free but Insurance pay”*	
Albania	Single level of Health Insurance which is mandatory in order to allow access to public hospitals. Additional voluntary Health Insurance in order to access private hospitals.
B & H	Public health care is organised at the cantonal level; with Insurance paid by employers to the Public Fund.
Croatia	Two levels of Health Insurance, basic and additional.
Czech Republic	
Estonia	There is a State-run Health Insurance.
Netherlands	Mandatory basic level of Health Insurance which is paid by everyone in employment. There are voluntary supplements available too.
Romania	National Public Health Insurance agency.
Serbia	Mandatory Social Health Insurance Scheme.
Slovakia	Mandatory Health Insurance, paid for by employer or State. 3 companies at present, 1 State run, 2 are private.
Slovenia	Health Insurance scheme run by the Government
Switzerland	Compulsory Basic level of Health Insurance. Additional ‘complementary’ health Insurance available too.
*“Pay at time of consultation”*	
Bulgaria	1.2E assuming individual paid contribution to National Health Fund. If not met contributions to National Health Fund then 10-15E.
Cyprus	Given inadequate Primary care physicians, if choose to see one privately will have to pay 50E.
Germany	10E per visit, or 40E per year and consultations are free.
Iceland	4E. Department of Health covers the rest via taxation.
Ireland	If not got a medical card (see above) then pay 60E. Some or all of this can be claimed from private Insurance scheme (50% population.
Norway	22E per visit, up to maximum of 260E per year including primary and secondary care appointments and prescription charges etc. In-patient stay is free. Government does collect income tax of which some goes to Department of Health.
Sweden	24E per visit, up to maximum of 180E per year.
*“Pay a certain amount/proportion”*	
Belgium	10% paid by patient, 90% paid by ‘social security’.
Finland	13.7E/visit for first 3 visits, then free.
France	23E at time of appointment but individual can claim back 70% of this from Social Security.
Greece	3-10E
Luxembourg	Individual pays 20% of 39.9E (ie 8E). Compulsory Public Health and Longterm Care Insurance means Government pays 80% of primary and secondary care consultation costs.

*B & H* Bosnia Herzegovina. *E* Euros

A recent taskforce of the European Respiratory Society (ERS) entitled European Initiative in Quality Management in Lung Cancer Care (EIQMLCC) provided evidence of the extent of variation in healthcare infrastructure, and also performed a feasibility study, the European Lung Cancer Audit (EuLuCA), collecting prospective data on patients with a new diagnosis of lung cancer [6]. Data collection is a key component in quality management and allows accurate evaluation of the epidemiological trends over time and a meaningful analysis of the variation in clinical care provision. However, despite this being a recommended approach [7], datasets currently developed for international use are likely to be beyond the ability of the majority of European countries to populate. This study aims to benchmark the European position in relation to the feasibility of collecting pan-European data by assessing the current practice with respect to data collection, and also to gauge the feasibility of, and interest in, a pan-European database for thoracic malignancy.

## Methods

Based on the network of lung cancer specialists established during the EIQMLCC taskforce who had participated in the EuLuCA project, a survey was distributed to 37 European countries in December 2014 (see Additional file 1). This survey was designed by the co-authors specifically to investigate the current status of data collection in Europe. The participants, all lung cancer physicians, gave written consent to participate in the project. They were also asked their opinion on 3 qualitative questions: what key challenges to prospective thoracic oncology data collection exist in their country; what is required to improve data acquisition and whether they would be willing to participate in a pan-European data collection programme.

## Results

Thirty-five of 37 countries returned completed surveys, a response rate of 95%. The participating countries are shown in Table 2; they comprise countries with a variety of socio-political structures and represent 64% of all European countries, as defined by the World Health Organisation. The countries within our cohort represent 68% of the population of Europe, or 93% of the population if Russia and the other former states of the USSR are excluded. Several countries of the former USSR fall within the region of central Asia, despite the WHO inclusion within Europe. Co-authors also sent examples of data collection forms, annual reports and the contact details of the individuals responsible for data collection in thoracic oncology in their country (Additional file 2).

**Table 2:** Basic features of data collection in 35 European countries

	Year est.	Mandatory	Consent	Form	Verbal	other	Data Completeness (%)	Year	Histo only	Clinical	C-R	DCO
Albania	2011	**No**	No				90%	2013	No	Yes	Yes	No
**Austria**	1969	Yes	No				Not available	N/A	**Yes**			
**Belgium**	2006	Yes	No				90–94	2013	No	Yes	Yes	
B & H	2004	Yes	No				59	2011	No	Yes	Yes	Yes
**Bulgaria**	1952	Yes	No				70–79	2011	**Yes**			
**Croatia**	1959	Yes	No				80–89	2013	**Yes**			
**Czech Rep**	1977	Yes	No				95–100	2013	No	Yes	Yes	No
**Denmark**	2000~	Yes	No				95–100	2013	No	Yes	Yes	Yes*
**Eng & Wales**	2003~	Yes	No				95–100	2013	No	Yes	Yes	No*
**Estonia**	1953	Yes	**Yes**	Yes			95–100	2011	No	No	Yes	Yes
**Finland**	1953	Yes	No				95–100	2012	No	Yes	Yes	Yes
**France**	1975	**No**	No				< 50%	2013	No*	No	Yes	No
**Germany**	1929	Yes	**Yes**	Yes			70–79	2013	No	Yes	Yes	Yes
**Greece**	2013	Yes	No				< 50%	2013	No	Yes	Yes	Yes
**Hungary**	1970~	Yes	No				70–79	2013	**Yes**			
**Iceland**	1955	Yes	No				95–100	2013	No	Yes-rarely	Yes	Yes-rarely
**Rep. Ireland**	1991	**No**	No				90–94	2012	No	No	Yes	Yes
Italy	1996	**No**	**Yes**		Yes		51	2013	**Yes**			
**Lithuania**	1984	Yes	No				95–100	2013	No	Yes-rarely	Yes	Yes-rarely
**Luxembourg**	2013	Yes	**Yes**			implicit	Not available	N/A	No	Yes	Yes	Yes
**Malta**	1957	Yes	No				95–100	2013	No	Yes	Yes	Yes-rarely
**Moldova**	1983	Yes	**Yes**	Yes			50–59	2012	**Yes**			
**Netherlands**	1989	**No**	**Yes**			implicit	95–97	2013	No	Yes	Yes	No
**Norway**	1953	Yes	No				97	2009	No	Yes	Yes	Yes
**Poland**	1952	Yes	No				80–89	2012	**Yes**			
**Portugal**	1988	Yes	No				60–69	2011	No	Yes	Yes	Yes
**Romania**	1981	Yes	No				< 66%	2011	No	Yes	Yes	Yes
**Scotland**	1958	Yes	No				95–100	2013	No	Yes	Yes	Yes
**Rep. Serbia**	1990	Yes	No				60–69	2013	No	No	Yes	Yes (PM)
**Slovakia**	1952	Yes	No				70–79	2008	No	No	Yes	Yes
**Slovenia**	1950	Yes	No				90–94	2010	No	Yes	Yes	Yes
Spain	1960	**No**	**Yes**	Yes			Not available	N/A	No	No	Yes	No
**Sweden**	1958	Yes	No				95–100	2013	No	No	Yes	No
Switzerland	1969	**No**	No				95–100	2013	No	Yes	Yes	Yes
**Turkey**	1993	**No**	No				< 50%	2009	No	Yes	Yes	No

Countries not in bold do not have a national dataset. *B&H* Bosnia and Herzegovina. *DCO* death certificate only. *N/A* not applicable. *PM* post-mortem only. Year est.; year that registry established

~ = Lung cancer specific data collection established. Histo only; only those patients with a histological or cytological diagnosis are recorded in the dataset. If no, then are cases confirmed on clinical grounds alone, or clinico-radiological grounds (C-R), and finally are cases included if the diagnosis of lung cancer is based on the death certificate only (DCO). Denmark; DCO*; accepted as diagnosis in National Cancer Registry, not in the National Lung Cancer Registry. England and Wales; DCO*; accepted as diagnosis in the National Cancer Registry not in the National Lung Cancer Audit. France; The Epithor surgical database would be histological confirmed cases only, the National Cancer Registry is not

### National data collection

Thirty countries collect data on a national level, with the majority using a national registry for all cancers. Several countries have a data collection programme for lung cancer in addition to a Cancer Registry, namely: Denmark, England and Wales, Germany, Hungary, The Netherlands, Norway, Scotland and Slovenia. Other countries have a specific thoracic surgery database, such as France, The Netherlands and Norway. There is no universal national data collection for lung cancer in Albania, Bosnia Herzegovina, Italy, Spain and Switzerland. The Albanian Respiratory Society has a register of lung cancer patients; described as a labour intensive paper record completed by senior doctors, and with limited clinical and survival data, with no formal analysis. There are two entities to Bosnia Herzegovina, the Federation of Bosnia Herzegovina and the Republic of Srpska. There is regional data collection for all cancers in Bosnia Herzegovina, with data collected electronically by the Federal Institute of Public Health. However, there is no data collection in the Republic of Srpska. In Italy there are 43 local cancer registries, of which 38 collect data on all cancer types, but 5 registries collect data on only certain cancer types, or for certain age groups. In contrast, there is national data collection for patients with mesothelioma in Italy, via the National Institute for Insurance against Accidents at Work (INAIL). The absence of national data collection in Spain and Switzerland is related to health care infrastructure. In Spain, there are 17 autonomous communities who control their own healthcare, and set their own agendas and priorities. In Switzerland, there are 26 cantons (regions) covered by 18 local cancer registries without a nationally defined dataset; currently only 15 of the 18 registries combine data at a national level.

### Basic features

Table 2 illustrates the basic features of these collection systems, showing the year cancer registration was established and where data collection is mandatory, and where patient consent is required. Data collection in half of our surveyed countries began between 1950 and 1980; with another nine countries starting between 1980 and 2000. Bosnia Herzegovina is the only country without a national data collection programme, but where data collection is mandatory at a regional level, in the Federation of Bosnia Herzegovina. Of those countries with a national programme for data collection, reporting is not mandatory in Germany, Rep. Ireland, the Netherlands and Turkey. Patient consent is required in 7 of the 35 countries, some at national and some at regional level. In some countries, such as Slovenia, Slovakia and Belgium, consent is not required for the national cancer registry, however patients need to consent for their data to be entered into the regional/hospital based lung cancer registries.

### Data completeness

Data completeness reflects the percentage of individuals with lung cancer reported in the regional or national datasets, as a percentage of the expected number of cases of lung cancer in that country, per year. It was quite variable. Seventeen of 35 countries reported completeness of > 90%. Bosnia Herzegovina, Greece, Italy, Moldova and Turkey reported data completeness of less than 60%, and in France although the data collected on patients in the Cancer Registry is below 50% complete; hospital records, collecting non-individualised data are 95–100% complete. Portugal, Romania and Rep. Serbia report data completeness between 60 and 69%, and Bulgaria, Germany, Hungary and Slovakia report completeness between 70 and 79% and Croatia and Poland report completeness between 80 and 89% (see Table 2). These data were based on the most up-to-date complete year of data collection, at the time of the survey, and are based on national or regional reports or publications. They were unavailable in three countries, Austria, Luxembourg and Spain.

### Data items

Twenty-eight countries include all patients diagnosed with histology, cytology or on the basis of clinical and radiological evidence. Seven countries (20%) collect data on only those patients with histologically confirmed disease, excluding other patients (Austria, Bulgaria, Croatia, Hungary (Koranyi pulmonology registry), Italy, Moldova and Poland). In contrast, some countries extend their denominator and also include those diagnosed on death certificate only, although some required confirmation at post-mortem.

Table 3 illustrates the data items collected by each country. Every country, except Austria, included date of diagnosis and sex, and all except Hungary and Republic of Serbia collected date of birth. These two countries record age at diagnosis instead. Every country records histology, and almost all use the WHO International Classification of Diseases for Oncology, 3rd edition. However in Denmark the SNOMED (Systematized Nomenclature of Medicine) system is used. Almost every country uses the ERS/ATS/IASLC system to classify adenocarcinoma [8]; exceptions were Germany, Malta, Moldova, Romania and Switzerland. Every country except Austria, Iceland and Malta record both TNM status and stage. Performance status (PS) was recorded in less than half of the countries surveyed. Belgium, Czech Republic, Denmark, England and Wales, France, Germany, Rep. Ireland, Luxembourg, Moldova, Norway, Poland, Scotland, Rep. Serbia and Sweden recorded PS in a national registry; whereas Albania, Italy and Spain record PS at a regional level. A similar number of countries record the smoking status of a patient. This information, however basic (current, ex, or never smoker), was recorded in: Austria, Croatia, Czech Republic, Denmark, Greece, Rep Ireland, Luxembourg, Moldova, Poland, Sweden and Turkey. Albania, Italy and Spain record smoking status at a regional level. The lung cancer registry of Slovenia, with 2/3 coverage, collects PS, smoking status, co-morbidity and molecular markers, although the national cancer registry does not. Socio-economic status (SES) was only recorded in five national datasets, namely: Denmark, England and Wales, Moldova, Poland and Scotland (calculated from patient’s postcode). Albania and Italy recorded SES at a regional level. Some countries record the occupation of an individual which could be used to infer their SES (Finland, the Republic of Ireland, Lithuania, Slovakia and Slovenia). In Norway, information on income and educational status can be obtained from Statistics Norway and the Norwegian patient register which can be linked to the Cancer Registry. It was not feasible to define which of these data items were mandatory in each country.

**Table 3:** Data items collected in current practice in 35 European countries

	Date dx	Histo	TNM	Stage	PS	Smoking	comorbid	SES	FEV1	KCO	EGFR	EML-4-ALK	MDT	1st line	2nd line	Last info date	Date of death
Albania							XXX			XXX	XXX	XXX	XXX	XXX	XXX	XXX	XXX
Austria	XXX		XXX	XXX	XXX		XXX	XXX	XXX	XXX	XXX	XXX		XXX	XXX	XXX	
Belgium						XXX	XXX	XXX	XXX	XXX	XXX	XXX					
B & H					XXX	XXX	XXX	XXX	XXX	XXX	XXX	XXX	XXX	XXX	XXX	XXX	
Bulgaria					XXX	XXX	XXX	XXX	XXX	XXX	XXX	XXX		XXX	XXX	XXX	
Croatia					XXX		XXX	XXX	XXX	XXX	XXX	XXX	XXX	XXX	XXX		
Czech Rep								XXX	XXX	XXX							
Denmark													OOO				
Eng & Wales						XXX				XXX	XXX	XXX					
Estonia					XXX	XXX	XXX	XXX	XXX	XXX	XXX	XXX	XXX			XXX	XXX
Finland					XXX	XXX	XXX	XXX	XXX	XXX	XXX	XXX	XXX				
France						XXX	XXX	XXX	XXX	XXX				XXX	XXX	XXX	
Germany						XXX	XXX	XXX	XXX	XXX		XXX					
Greece					XXX		XXX	XXX	XXX	XXX	XXX	XXX	XXX		XXX	XXX	XXX
Hungary					XXX	XXX	XXX	XXX	XXX	XXX	XXX	XXX			XXX	XXX	XXX
Iceland			XXX	XXX	XXX	XXX	XXX	XXX	XXX	XXX	XXX	XXX	XXX	XXX	XXX	XXX	
Rep. Ireland							XXX	XXX	XXX	XXX	XXX	XXX					
Italy									XXX	XXX			XXX				
Lithuania					XXX	XXX	XXX	XXX	XXX	XXX	XXX	XXX	XXX	XXX	XXX	XXX	
Luxembourg								XXX	XXX	XXX					XXX		
Malta			XXX	XXX	XXX	XXX	XXX	XXX	XXX	XXX	XXX	XXX	XXX	XXX	XXX		
Moldova									XXX	XXX	XXX	XXX					
Netherlands					XXX	XXX	OOO	XXX	XXX	XXX			XXX		XXX	XXX	
Norway						XXX	XXX	XXX	XXX	XXX							
Poland							XXX		XXX	XXX		XXX					
Portugal					XXX	XXX		XXX	XXX	XXX		XXX					
Romania					XXX	XXX	XXX	XXX	XXX	XXX	XXX	XXX	XXX			XXX	
Scotland						XXX	XXX		XXX	XXX		XXX			XXX	XXX	
Rep. Serbia						XXX	XXX	XXX	XXX	XXX		XXX			XXX	XXX	
Slovakia					XXX	XXX	XXX	XXX	XXX	XXX	XXX	XXX	XXX		XXX		
Slovenia					XXX	XXX	XXX	XXX	XXX	XXX	XXX	XXX	XXX		XXX	XXX	
Spain								XXX									
Sweden							XXX	XXX	XXX	XXX		XXX			XXX	XXX	
Switzerland					XXX	XXX	XXX	XXX	XXX	XXX	XXX	XXX	XXX		XXX		
Turkey					XXX			XXX	XXX	XXX	XXX	XXX	XXX	XXX	XXX		

Legend: White box means data item is collected. XXX means data item is not currently collected. OOO means data item only sometimes collected

*B&H* Bosnia Herzegovina. *Date dx* date of diagnosis. *Histo* histological subtype. *PS* performance status. *Comorbid* co-morbidity. *SES* socioeconomic status. *KCO* transfer factor. *MDT* multidisciplinary team. 1st line and 2nd line refer to treatment given. Last info date = follow-up data recorded up to point of death or censorship for annual report

Lung function, either spirometry or transfer factor, was only recorded in Albania, Denmark, England and Wales and at a regional level in Spain. Co-morbidity was only recorded in 9 countries as routine practice, although the majority did report this feature in research projects. Table 4 illustrates the different measures of co-morbidity, performance status and quality of life (QOL) used across Europe. The Charlson Index [9] and ACE-27 [10] were the most popular methods for recording co-morbid state. Denmark is the only country to record data on quality of life (QOL) at diagnosis and after treatment. In the Czech Republic, data on QOL is recorded at diagnosis, and the majority of countries record QOL in the research setting only.

**Table 4:** Illustrates the variation in methods used to record performance status, co-morbidity and quality of life

	Performance status		Co-morbidity				Quality of Life (QOL)				
	ECOG/WHO	Karnofsky	Charlson	ACE 27	Specific	Other	EORTC	FACT-G	SF-36	FACIT	Other
Albania	Yes	Yes			Yes						None
Austria	Yes					Research					Research
Belgium	Yes		Yes				Yes*				
B & H	Yes		Yes				Yes				
Bulgaria	Yes	Yes**			Yes		Yes*				
Croatia	Yes	Yes			Yes						None
Czech Rep	Yes					None	Yes	Yes			
Denmark	Yes		Yes		Yes		Yes				EORTC LC13
Eng & Wales	Yes		Yes	Yes	No*		Yes*		Yes*		
Estonia	Yes*	Yes*				None					None
Finland	Yes	Yes**			Yes						Research
France	Yes		Yes				Yes				
Germany	Yes	Yes	Yes*				Yes*				
Greece	Yes	Yes	Yes*	Yes*	Yes*		Yes*	Yes*	Yes*	Yes*	
Hungary	Yes	Yes				None					
Iceland	Yes				Yes		Yes*				
Rep. Ireland	Yes					None	Yes*		Yes*		
Italy	Yes	Yes			Yes		Yes				
Lithuania	Yes					None					None
Luxembourg	Yes		Yes								None
Malta	Yes	Yes			Yes		Yes				
Moldova		Yes				None			Yes		
Netherlands	Yes		Yes				Yes*				
Norway	Yes		Yes*				Yes	Yes**	Yes**	Yes**	
Poland	Yes				Yes		Yes				
Portugal	Yes	Yes**	Yes				Yes*				
Romania	Yes					None					None
Scotland	Yes					SLCFCSS	Yes*				
Rep. Serbia	Yes				Yes**		Yes**				
Slovakia	Yes	Yes			Yes		Yes*				
Slovenia	Yes				Yes						None
Spain	Yes				Yes						None
Sweden	Yes					No**	Yes				
Switzerland		Yes	Yes								Variation
Turkey	Yes	Yes				None	Yes*	Yes*			

Legend: Charlson = Charlson Index [9]

*ACE 27* Adult Co-morbidity Evaluation score [10], *SLCFCSS* Scottish Lung Cancer Forum Co-morbidity Scoring System [45], *Specific* specific co-morbid diseases are recorded, *EORTC QLQ-C30* European Organisation for Research and Treatment of Cancer Quality of Life Questionnaire [46], *FACT-G* Functional Assessment Cancer Therapy-General [47], *SF-36* Short Form-36 [48], *FACIT* Functional Assessment Chronic Illness Therapy [49]

Yes* = research/clinical trials only

Yes** = infrequently

No* = no longer used

No** = Co-morbidity recorded only if it prevented planned treatment

Recording the treatment given to a patient was not universal; neither was confirming discussion at a multi-disciplinary team (MDT) meeting. In fact, it appears MDTs are not mandatory in Romania; they exist in certain centres, but there is no strict guidance as to their composition. Almost every country recorded a date of death, the only exceptions at the time of the survey were; Albania, Estonia, Greece and Hungary.

### Qualitative results

**Fig. 1:**
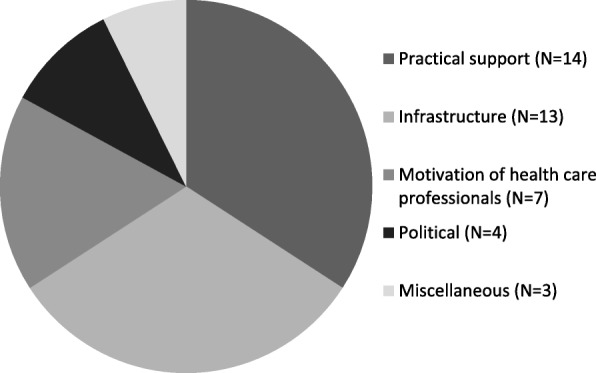
Reported problems in achieving national data collection in 28 European countries. Legend: *Practical support* refers to the need for more funding and staff to support data collection. *Infrastructure* includes regional not national datasets, and those countries where private and university hospitals are not linked, or respiratory and oncology hospitals that work independently. It also includes the absence of a single patient identifier, and also those countries without electronic transfer of data. *Political will* was stated by 1 co-author as was concern regarding legal requirements and issues of patient consent by a further 3 co-authors. *Miscellaneous* includes quite specific difficulties encountered in three countries. One co-author stated an historical lack of interest in epidemiology as a whole as a barrier to better data collection. Three languages are spoken in one country and in another, patients are often treated abroad, which makes evaluating treatment outcomes and follow-up very difficult. Seven countries stated there were no difficulties in collecting data at a national level

There were a number of themes which emerged when the national representatives were asked what the key challenges were to universal data collection in their own country. Healthcare infrastructure with closer links between private and public sectors was cited as a requirement to facilitate a common hospital dataset with a unique patient identifier. Technological limitations, with no electronic patient record, and inadequate personnel to support a national dataset were issues for some. Motivation and education of clinicians was also identified as a barrier to universal uptake. Finally there was an acknowledgement from some that funding would be the key challenge, and a concern regarding the legality of a national patient dataset (Figure 1).

However, there was a very clear positive response towards the idea of a pan-European dataset of thoracic oncology. Twenty of the participants gave a definite positive response to this aspiration (57%), and a further 5 (14%) confirmed they would be keen if there were enough resources and assuming this did not result in duplication of work. Another 5 (14%) participants would support this work if there was national agreement, or it was made mandatory. One participant was quite neutral, and only 2 (6%) were opposed to the idea of a pan-European dataset.

## Discussion

### Main findings

The main finding of this study is that data are being collected in the majority of European countries, but the nature, extent, and hence the usefulness of these data varies considerably. Surprisingly some basic demographic items as well as important factors predictive of outcome were omitted in some datasets, and do not form part of the European Network of Cancer Registries’ (ENCR) recommendations [11, 12]. Socio-economic status and performance status are two of the most important predictors of outcome [13–17], yet data recording and completeness of these data items was highly variable. The majority of countries already use computerised reporting, with linkage to demographic information resources which allows survival analyses to be performed. However, in Albania, Estonia, Greece, Hungary, Malta and Romania these survival data are not collected, and the use of paper records remains current practice in Albania, Croatia, Lithuania and Romania. Many countries have a cancer registry, with good levels of data completeness, but they often lack the level of clinical detail required for evaluating quality management in thoracic oncology care.

We identified significant and important differences in the denominator used. The exclusion of cases which lack histological confirmation will make comparisons difficult because the size of the denominator will depend on the histological confirmation rate. Furthermore those countries that allow inclusion of death certificate only cases will have a comparatively poor outcome. It is clear from these two findings (variation in data items collected and denominator) that there needs to be agreement between interested parties (such as the ENCR, respiratory, oncology and surgical societies) on both patients included and the list of data items with specific definitions, ensuring feasibility of data collection in each country.

Another important finding from this survey is that within this selected group of clinicians, with only two exceptions, there was support to create a pan-European core dataset for thoracic oncology. This is an important area of development and one which demands the involvement of committed clinicians representing all disciplines.

### Strengths and weaknesses

The main strength of this study is the high level of participation including 35 European countries. This has generated a comprehensive description of current practice in data collection in thoracic oncology from all areas of Europe. It is difficult to verify the self-reported data completeness levels given several countries do not report their data quality, and in those countries where data collection occurs at the local level, it is difficult to ensure we have correctly reported the data items used. A survey can only ever be descriptive and could be open to bias, but all the national representatives are physicians involved in thoracic oncology care and there was no financial remuneration or pharmaceutical involvement which could have influenced the results. We therefore believe this to be an accurate reflection of current practice across Europe and the first survey to provide a pan-European picture.

### Comparison with published data

There is very little published literature regarding the variation in data collection across Europe. However, in the past 25 years, the use of data to evaluate lung cancer care and make comparisons between areas of the world has become more common. It was in 1989, during his presidency of the European Union that Francois Mitterrand initiated a health programme on cancer prevention and patient information from which the EUROCARE papers have all arisen [2, 4, 18]. The EUROCARE studies are an excellent example of how data have been used to assess health outcomes, and the results have led to a change in healthcare funding and structure. Although the EUROCARE-5 database contains approximately 22 million patients, from 26 countries [19], the actual coverage within some of these countries is below 1% population, which can introduce geographical bias [20, 21]. And there is evidence that some countries have incomplete follow-up data, which for a cancer with a poor prognosis, such as lung, can lead to falsely reassuring survival results [22]. Furthermore, these studies lack the level of clinical detail, such as performance status and stage, which are required to make direct clinical comparisons between countries. There is also variation between countries and their Registries as to whether they rely on histologically confirmed cases only, and whether they accept individuals diagnosed by death certificate only. In both situations, the cohort of patients with cancer will be different for those Registries who accept patients based on a clinical or radiological diagnosis or post-mortem compared with those Registries which do not. This is particularly relevant for cancers with a short survival like lung, and could create a systematic bias causing survival figures to appear better than they are for the whole population.

The National Lung Cancer Audit (NLCA) in England was established in 2004, to allow prospective data collection on all patients given a diagnosis of lung cancer and mesothelioma. This dataset, validated in 2009 [17], has shown a year on year improvement in both data acquisition and data completeness and has been used to assess inequalities in outcome based on patient and hospital features [23–30]. There has also been a demonstrable improvement in key quality performance indicators over the lifetime of the NLCA [31, 32]. Other European countries have developed similar systems for data collection and used these data to evaluate current practice and address any inequality that may be seen, including Denmark, Norway and The Netherlands [33–38]. The Danish Lung Cancer Group wrote clinical guidelines in 1998, and started prospective data collection in 2000. They have been able to demonstrate that the use of data collection to monitor guideline adherence, audit performance at the local level and benchmark standards nationally, has led to an objective improvement in lung cancer outcome measures [39].

The International Cancer Benchmarking Project (ICPB) was set up in 2009, linking established cancer registration programmes in 6 countries across 3 continents, in order to look at cancer outcomes. It is thus limited to only a few countries. Lung cancer survival has been studied within this group and variation described, with Denmark and the UK observed to have lower survival compared to Canada, Sweden, Norway and Australia [40]. Furthermore, the International Consortium for Health Outcomes Measurement (ICHOM) published a comprehensive revised data collection reference guide in April 2015. Their aim is to create a standardised set of measurements, which can be used to compare performance between countries, and allow clinicians to learn from each other, and improve the provision of lung cancer care [41]. Both the ICBP and ICHOM require a level of detail of data collection that is likely to be beyond the capability of many European countries for the foreseeable future; what is required is a pragmatic solution.

The expansion of the European Union, and greater freedom of movement across borders, has led to European ministers beginning to address the issue of collaboration between national health services [42]. However, many European countries have healthcare systems that have evolved as the political situation changes, for example the war of independence in Croatia lead to significant damage to the previously thriving cancer services [43]. It is this variation in socio-political stability that creates widely disparate healthcare systems. In order to understand variation in lung cancer outcome, one must acknowledge the variation in infrastructure, facilities, and treatments which are available.

In 2006 Ludwig, an Austrian oncologist, recommended a pan-European action plan on cancer, with bench-marking of the quality and effectiveness of the various healthcare systems [44]. This survey could form the background upon which a pan-European core dataset on thoracic oncology is built. The mechanism would involve an iterative approach based on what is feasible in each country, slowly building a more detailed dataset; the vehicle could be the network already established by the ERS Taskforce.

## Conclusion

Improving the standard of care for our patients should be the aim of every clinician involved in thoracic oncology care, and in order to evaluate different practices across Europe we need to be able to understand the political and economic setting in which it is based. Data collection can play an important role in evaluating medical practice and ensuring that whilst a cure for lung cancer and mesothelioma may not be on the horizon, the delivery of best available treatments should be realistic. Data collection itself relies on adequate infrastructure, dedicated personnel, and financial investment in the information technology to support large scale datasets. The results of this study have shown that there is genuine interest in pan-European data collection and a pressing need to develop a standardised dataset that is feasible for all to collect. To this end, a European Respiratory Society taskforce is developing both an essential (redacted) and minimum dataset. This is an important project upon which to build as it will allow meaningful analyses across Europe that can be used to drive improvements in care for our patients.

## Additional files

Additional file 1: Survey for EuLuCA representatives. (DOCX 20 kb)

Additional file 2: Additional information provided by lung cancer physicians regarding thoracic oncology data collection in 35 European countries. (DOCX 22 kb)

## Abbreviations

ACE-27: Adult Co-morbidity Evaluation-27
ATS: American Thoracic Society
EIQMLCC: European Initiative in Quality Management in Lung Cancer Care
ENCR: European Network of Cancer Registries
ERS: European Respiratory Society
EuLuCA: European Lung Cancer Audit
IASLC: International Association Staging in Lung Cancer
ICBP: International Cancer Benchmarking Project
ICHOM: International Consortium for Health Outcomes Measurement
INAIL: Italian National Institute against accidents at work
MDT: Multi-Disciplinary Team
NLCA: National Lung Cancer Audit
PS: Performance Status
QOL: Quality of Life
SES: Socio-Economic Status
SNOMED: Systematised Nomenclature of Medicine
TNM: Tumour Node Metastasis
WHO: World Health Organisation
