# Supplementary material for: Achieving Thoracic Oncology data collection in Europe: a precursor study in 35 Countries
Source: BMC Cancer. 2018 Nov 20;18:1144. doi: 10.1186/s12885-018-5009-y (PMC6247748; doi:10.1186/s12885-018-5009-y)
Supplement: Supplementary file 1 — Survey for EuLuCA representatives. (DOCX 20 kb) [file 12885_2018_5009_MOESM1_ESM.docx]

Additional file 1

**EuLuCA review article**

**Survey for EuLuCA representatives**

***Please answer for your country as a whole***.

If there is variation within your country, please try to describe this.

**Organisation of national cancer registration in thoracic oncology**

**1**. Does your country collect any data on lung cancer patients at a national level?______________

***If yes***; please choose the most appropriate answer(s)

a. Government or ministry of health

b. National societies

If so, please name them._____________________________

c. Network of lung cancer centres

d. Tumour biobank data registry

e. Cancer data registry

f. Others (please describe):

If so, is this data collection :

1) for all cancers

2) restricted to lung cancer only?

Please record the contact details and preferably contact person(s) of all national registries:

***If there is no data collection on a national level***

Is there data collection on a regional and/or institutional level? (please describe)

**2**. Do you collect any data on patients with mesothelioma?

***If yes***; please choose the most appropriate answer(s)

a. Government or ministry of health

b. National societies

If so, please name them._____________________________

c. Network of lung cancer centres

d. Tumour biobank data registry

e. Cancer data registry

f. Others (please describe):

If so, is this data collection:

1) for all cancers

2) restricted to mesothelioma only?

Please record the contact details and preferably contact person(s) of all national registries:

***If there is no data collection on a national level***

Is there data collection on a regional and/or institutional level? (please describe)

**Infrastructure of national cancer registration in thoracic oncology**

We anticipate there will be variation within your country regarding the following questions, so if you are able to describe this it would be very helpful.

**3**. Who collects/reports and types in the data? (multiple answers are possible)

a. data administrators

b. nurses

c. junior doctors

d. senior doctors/consultant in charge of care

e. Others (please name):

Description of variation:

**4**. How are data collected in your country? (multiple answers are possible)?

a. paper record

b. computer linked to hospital information system

c. computer linked to national registry/audit programme

d. Others (please name):

**5**. If computerised programmes are used; what programmes or operating systems are these?

Please give as much detail as you can:

**Definitions for national cancer registration in thoracic oncology**

**6**. Do you *only* record patients with a histologically (or cytologically) confirmed diagnosis?________

***If no***; do you include;

a. patients diagnosed with lung cancer on a clinical basis

b. patients diagnosed with lung cancer on a clinical ***and*** radiological basis?

c. patients with lung cancer recorded on the death certificate without any prior investigations?

**7**. Is there a published catalogue of data parameter definitions used in your country?

**If yes,** please send us these catalogues as an attachment.

**Requirements for national cancer registration in thoracic oncology**

**8**. Is it mandatory within your country to collect data on individuals with lung cancer?

a. yes

b. no

**9**. Is there a legal requirement for patients to give their consent for data to be recorded?

a. yes

b. no

**If yes**, how is this done?

a. specific form/letter signed by the patient

b. verbal consent which is documented in clinical notes

c. Other (please describe):

**10**. If these data are used for research, is approval by an ethical board/committee required beforehand?

a. yes

b. no

**Completeness of national cancer registration in thoracic oncology**

**11**. Do you know how ‘complete’ the lung cancer registration system in your country is?

In other words, do you know *what proportion of patients estimated to have lung cancer in your country* *are included in the national registration system*?

Please state the proportion as percentage for 2013 (or the last completed year):

*Year:* 20__

a.95-100%

b.90-94%

c.80-89%

d.70-79%

e.60-69%

f.50-59%

g. <50%

Exact percentage (if known): _____%

**12**. Are survival data linked to the national cancer registration system in your country, in order that individual (and trends in) survival can be calculated?

a. yes

b. no

**If yes,** how are survival data obtained by the national cancer registry (multiple answers possible):

a. survival data obtained by linkage to local/regional/national registration offices/authorities databases

b. survival data obtained by linkage to other national/regional cancer registries

c. queries of survival status from other professional caretakers (i.e. general practitioners, other hospitals)

d. queries of survival status directly from patients/relatives/next of kin

e. Others (please name):

**13*.*** What are the data collected in the national cancer registry used for?

For example;

a. are they published in annual reports?

b. are they used by the Government/Ministry of Health to rationalise healthcare in your country?

c. are the results available to individual institutions/clinicians?

d. can they be used in research projects?

e. others (please name):

Please provide links to, or attach the latest version of the annual report of your national cancer registry if these exist.

**Questions for the future**

14. What are the key challenges that prevent universal data collection in your country?

15. If you could write a ‘wish list’ of things that are needed in order to achieve universal data collection in thoracic oncology in your country, what would they be?

16. If a Pan-European data collection programme was created for thoracic oncology, would you and your colleagues be keen to participate?

**Data fields collected for lung cancer**

**17**. We have made a table of potential data fields/parameters that your national cancer registry may collect. Please tick yes or no depending if these data fields/parameters are included in your national data registry or not.

However, your national cancer registry may well collect additional data:

**If there is an electronic or printed document listing and defining all included data fields/parameters of your national cancer registry, please send us this as an email attachment.**

***Only if*** a document of this kind does NOT exist in your country, we would kindly like to ask you to type in the additionally collected parameters under ‘Others (please list):’ into the table below.

| ***Collected data field/parameter in your national cancer registry*** | YES | NO |
| --- | --- | --- |
| **Demographic data** | | |
| -Date of birth |  |  |
| -Age |  |  |
| -Sex |  |  |
| -Others (please list): | | |
|  | | |
| **Baseline data at time of diagnosis** | | |
| -date of diagnosis |  |  |
| -Histological subtype |  |  |
| -TNM status |  |  |
| -Stage |  |  |
| -Performance status |  |  |
| -Smoking status (current, ex, never. Pack years?) |  |  |
| -comorbidities and/or comorbidity index |  |  |
| -socio-economic status |  |  |
| -Lung function; FEV1 (absolute and/or percent predicted) |  |  |
| Gas transfer (absolute and/or percent predicted) |  |  |
| -Others (please list): | | |
|  | | |
| **Molecular pathology data** | | |
| -EGFR mutation status |  |  |
| -EML-4-ALK translocation status |  |  |
| -Others (please list): | | |
|  |  |  |
| **MDT discussion** (Yes/no and date) |  |  |
|  |  |  |
| **Treatment data** | | |
| -First line treatment plan (modality and date) |  |  |
| -Second line treatment plan (modality and date) |  |  |
| -Others (please list): | | |
|  | | |
| **Follow-up data** | | |
| -last info date |  |  |
| -date of death |  |  |
| -Others (please list): | | |
|  | | |
| **Quality of Life (QOL)** | | |
| -Is any record made of QOL at diagnosis? |  |  |
| -Is any record made of QOL after treatment? |  |  |
| -Others (please list): | | |

**18**. What cytological/histological classification system for lung cancer do you use in your country (multiple answers possible):

a. WHO International Classification of Diseases for Oncology, 3^rd^ Edition (ICD-O-3)

b. Others (please name):

If there is variation in your country, please describe:

**19**. *In addition*, do you use the ERS/ATS/IASLC classification system for Adenocarcinoma in your country?

a. yes

b. no

If there is variation in your country, please describe:

**20**. What staging system for lung cancer do you use in your country? (multiple answers possible)

a. UICC 7

b. Others (please name):

If there is variation in your country, please describe:

**21**. How do you assess performance status in your country? (multiple answers possible)

a. ECOG/WHO classification

b. Karnofsky index

c. Others (please name):

d. not at all

If there is variation in your country, please describe:

**22**. How do you assess co-morbidity in your country? (multiple answers possible)

a. Charlson Co-morbidity Index

b. ACE 27 score

c. Another co-morbidity index (please state):

d. Documentation of selected comorbidities

e. Others (please name):

f. not at all

If there is variation in your country, please describe:

**23**. How do you assess quality of life in your country?

a. EORTC QLQ-C30

b. FACT-G (Functional Assessment Cancer Therapy-General)

c. SF-36 (Short Form - 36)

d. FACIT (Functional Assessment Chronic Illness Therapy)

e. Others (please name):

f. not at all

If there is variation in your country, please describe
