# Supplementary material for: Achieving Thoracic Oncology data collection in Europe: a precursor study in 35 Countries
Source: BMC Cancer. 2018 Nov 20;18:1144. doi: 10.1186/s12885-018-5009-y (PMC6247748; doi:10.1186/s12885-018-5009-y)
Supplement: Supplementary file 2 — Additional information provided by lung cancer physicians regarding thoracic oncology data collection in 35 European countries. (DOCX 22 kb) [file 12885_2018_5009_MOESM2_ESM.docx]

Additional file 2: Additional information provided by participants

| **Country** | **Additional information, websites and contact details etc** |
| --- | --- |
| Albania | Albanian Respiratory Society’s own lung cancer register  No Chemo or radiotherapy data, and no survival data. |
| Austria | Prof Klepetko, meduniwien. |
| Belgium | National Cancer Registry; <http://www.kankerregister.org/Home>  Mrs E Van Eycken; [Elizabeth.vaneycken@kankerrregister.org](mailto:Elizabeth.vaneycken@kankerrregister.org) |
| Bosnia Herzegovina | Federation B&H; Marija Zeljko; [m.zeljko@zzjzfbih.ba](mailto:m.zeljko@zzjzfbih.ba) |
| Bulgaria | [ndimitrova@sbaloncoloy.bg](mailto:ndimitrova@sbaloncoloy.bg) |
| Croatia | http://hzjz.hr/sluzbe/sluzba-za-epidemiologiju/odjel-za-nadzor-i-istrazivanje-ne-zaraznih-bolesti/odsjek-za-zlocudne-bolesti-s-registrom-za-rak/ |
| Czech Republic | National Oncology Registry, within Institute of Health Information and statistics (UZIS).  http://www.uzis.cz/system/files/zpok_nor_010_20140101.pdf  Dr Dusek Ladislav; [ladislav.dusek@uzis.cz](mailto:ladislav.dusek@uzis.cz)  TULUNG, registry of patients with NSCLC treated with targeted agents according to biomarkers; http://tulung.registry.cz/index.php?pg=vysledky  Dr Hejduk Karel; hejduk@iba.muni.cz |
| Denmark | Danish Lung Cancer Group  Dr Torben Riis Rasmussen, chariman  Dept Pul Medicine, Aarhus University.  Danish Cancer biobank  Dr Estrid Hogdall,  Dept of pathology, Herlev Hospital.  Danish Lung Cancer Registry  Dr Erik Jakobsen  Dept Thoracic Surgery, Odense University Hospital.  National Danish Cancer Registry  Cancerregisteret,  Statens serum Institut, Copenhagen  National Pathology Data bank  Dr Beth Bjerregard  Part of pathology, Herlev Hospital. |
| England and Wales | National Lung cancer Audit  Dr Ian Woolhouse; [ian.woolhouse@uhb.nhs.uk](mailto:ian.woolhouse@uhb.nhs.uk)  National Cancer registration Service  Jem Rashbass; [jem.rashbass@phe.gov.uk](mailto:jem.rashbass@phe.gov.uk)  Data parameters;  <https://www.rcplondon.ac.uk/sites/default/files/cosd_dataset_v6_0_lung_core_items.xls>  Annual report;  <https://www.rcplondon.ac.uk/sites/default/files/nlca_annual_report_2014.pdf> |
| **Country** | **Additional information, websites and contact details etc** |
| Estonia | Estonian Cancer Registry,  National institute for Health Development, Tallinn.  Margit.magi@tai.ee |
| Finland | Finnish cancer Registry,  Institute for Statistical and Epidemiological Cancer Research  [www.cancerregistry.fi](http://www.cancerregistry.fi) |
| France | French Society of Thoracic surgery collects data on surgical cases only (EPITHOR).  National Institute against cancer (INCa)  Mesothelioma data is collected by Institut National contre le Cancer, Boulogne-Billancourt. |
| Germany | Arbeitsgemeinschaft Deutscher Tumorzentren (ADT)  (Association of German Tumour Registries)  <http://www.tumorzentren.de>  [adt@tumorzentren.de](mailto:adt@tumorzentren.de)  Gesellschaft der epidemiologischen Krebsregister in Deutschland (GEKID)  (Society of Epidemiological Cancer Registries in Germany)  http://www.gekid.de  Onkozert  (Data from certified lung cancer centres of the German Pneumology Society, the German Thoracic Surgery Society and the German Cancer Society)  <http://www.onkozert.de/>  [info@onkozert.de](mailto:info@onkozert.de)  Statistisches Bundesamt  <https://www.destatis.de/DE/Startseite.html>  Deusches Zentrum fur Lungenforschung  Tumour biobank within the German centre for lung research  Dr Megan Grether, Giessen  [Megan.grether@innere.med.uni-giessen.de](mailto:Megan.grether@innere.med.uni-giessen.de)  <http://www.dzl.de> |
| Greece | Hellenic cancer registry  Dr Lia Tzala; [ean@keelpno.gr](mailto:ean@keelpno.gr) |
| Hungary | National Cancer registry, Hungarian Cancer registry  <http://ercim-news.ercim.eu/en68/rd/new-hungarian-national-cancer-registry>  National Koranyi Institute of Pulmonology and Tuberculosis,  Prof I Horvath, Budapest;  [ildiko.horvath@koranyi.hu](mailto:ildiko.horvath@koranyi.hu)  <http://www.koranyi.hu/tartalom/bulletin/Evkonyv2014.pdf> |
| Iceland | Icelandic Cancer Registry,  Dr J Jonasson; Reykjavik  <http://www.krabbameinsskra.is/indexen.jsp?id=summary> |
| **Country** | **Additional information, websites and contact details etc** |
| Rep. of Ireland | The National Cancer Control programme, Dept of Health  The National Cancer Registry  Cork. [info@ncri.ie](mailto:info@ncri.ie)  <http://www.ncri.ie/sites/ncri/files/pubs/NCRReport_19942012_Dec2014.pdf> |
| Italy | National Mesothelioma Registry, coordinated by National Institute for Insurance against Accidents at work (INAIL).  Email; [r.dml@inail.it](mailto:r.dml@inail.it) |
| Lithuania | Lithuanian Cancer Registry  [www.nvi.lt](http://www.nvi.lt) |
| Luxembourg | Registre National du Cancer,  Luxembourg Institute of Health,  www.rnc.lu |
| Malta | Ms Rita Micallef; [rita.t.micallef@gov.mt](mailto:rita.t.micallef@gov.mt)  <https://ehealth.gov.mt/healthportal/Chief_Medical_Officer/healthinfo_research/registries/cancers.aspx> |
| Moldova | Cancer Registry of Moldova by Oncological Institute of Moldova  <http://onco.md/> |
| The Netherlands | Cancer data registry; [www.iknl.nl](http://www.iknl.nl)  Tumour biobank data registry; [www.palga.nl](http://www.palga.nl)  Surgical audit; [www.clinicalaudit.nl](http://www.clinicalaudit.nl)  Radiotherapy audit: [www.clinicalaudit.nl](http://www.clinicalaudit.nl) |
| Norway | Cancer Registry of Norway  The main registry contains 5 national cancer quality registries, lung cancer is one. Dr Lars Fjellbirkeland is leader of Reference group.  <http://www.kreftregisteret.no/en/General/Publications/Cancer-in-Norway/>  <http://www.kreftregisteret.no/no/Generelt/Publikasjoner/Arsrapporter/Arsrapport-2013/>  Society of Thoracic Surgeons; Registry of all surgical patients. |
| Poland | Polish Cancer Registry  <http://onkologia.org.pl/> |
| Portugal | Registo Oncológico Regional (ROR):  Dr L. Correia, North Lisbon Hospital Centre, coordinator for the South ROR/RORS, who can provide links with other ROR centres.  [Lucorreia.mail@sapo.pt](mailto:Lucorreia.mail@sapo.pt)  To access copy of electronic database contact: Dr A. Miranda [amiranda@ipolisboa.min-saude.pt](mailto:amiranda@ipolisboa.min-saude.pt)  [www.dgs.pt](http://www.dgs.pt)  [www.ror-sul.org.pt](http://www.ror-sul.org.pt)  Sociedade Portuguesa de Pneumologia;  [www.sppneumologia.pt](http://www.sppneumologia.pt)  [sppneumologia@mail.telepac.pt](mailto:sppneumologia@mail.telepac.pt)  Tumour biobank started December 2013  <http://www.rnbt.org> |
| **Country** | **Additional information, websites and contact details etc** |
| Romania | National Cancer Registry, managed by Centre for Medical Statistics |
| Scotland | Director Scottish Cancer Registry,  Dr David Brewster: [david.brewster@nhs.net](mailto:david.brewster@nhs.net)  Dr Robert Milroy: [robert.milroy@ggc.scot.nhs.uk](mailto:robert.milroy@ggc.scot.nhs.uk)  Chair of Scottish Lung Cancer Forum, |
| Rep. Serbia | Republic Statistic Institute, and Republic Institute of Public Health  [Dragan_miljus@batut.org.rs](mailto:Dragan_miljus@batut.org.rs)  With respect to Mesothelioma; data collected in registries at 2 major institutions. University Hospital of Pulmonology, Belgrade and Institute of Pulmonary Diseases, Vojvodina. |
| Slovakia | National health Information Center,  National cancer registry of Slovakia,  Bratislava.  <http://www.nczisk.sk/Pages/default.aspx>  Dr S Diba; [diba@nczisk.sk](mailto:diba@nczisk.sk)  <http://www.nczisk.sk/Registre/Narodne-zdravotne-registre/Pages/Narodny-onkologicky-regitser.aspx> |
| Slovenia | Cancer Registry of Republic of Slovenia; [www.slora.si](http://www.slora.si)  Ljubljana. Established in 1950.  Maja Primic Zakelj, Head of Registry.  Lung Cancer Registry of the University Clinic of Golnik (UCG). Established in 2010 and collecting data on 2/3 all patients with thoracic malignancies in Slovenia. Transfer of data between both registries exists. |
| Spain | No national data collection |
| Sweden |  |
| Switzerland | <http://www.nicer.org>  National Institute for Cancer Epidemiology and Registration |
| Turkey | Cancer Control Dept, Turkish ministry of Health.  [www.kanser.gov.tr](http://www.kanser.gov.tr)  Data parameters; <http://kanser.gov.tr/daire-faaliyetleri/kanser-kayitciligi.html> |
